# Conference at Hull

**Published:** 1918-11-02

**Authors:** 


### Conference at Hull.

THE conference of representatives of the Friendly Societies, trade unions, and kindred organisations held recently at the Royal Institution, Hull, was called by the Local Branch of the Council for Combating Venereal Diseases. The Lord Mayor of the city, Councillor H. Johnson, J.P., was supported by Dr. Harrison, physician in charge of the clinic at the Hull Infirmary; the Rev. E. A. Berry (Vicar of Drypool), Dr. Verdon, Dr. G. W. Lilley, J.P., Dr. Ethel Townend, Mr. J. Lewenstein, clerk to the Hull Insurance Committee and Local Hon. Sec. to the Branch, Mr. E. Horner (National Union of Dockers), Rev. Dr. Dalton, Canon Scott Ram, and several Poor-Law Guardians.

The Lord Mayor stated that the Munitions Department was willing to allow lectures to be given at various works during working hours on the subject.

Dr. E. Harrison explained the present system of treatment, and stated that 450 cases had been dealt with at the clinic during the past nine months. He regretted to state that many patients ceased to attend the clinic after being in attendance some time "because they felt better," although to receive full benefit they should attend until the physician definitely told them that further visits were unnecessary.

Mr. E. Horner discussed the Labour aspect, and proposed a resolution urging the necessity of educating the people in the means for the most effective treatment, and asking large employers of labour to allow competent persons to lecture to their workers during the employers' time. Mr. W. Sims, on behalf of the Friendly Societies Council, seconded and Mr. F. Potter supported the resolution, which was carried unanimously. A number of social workers in the city are quite enthusiastic over the work. Mr. C. M. Clark, and Sister Emmeline Downing, of the Hull Vigilance Association, have had a course of instruction at Oxford to enable them to lecture to the public. So Hull is making headway.

